# Probing a CNN–BiLSTM–Attention-Based Approach to Solve Order Remaining Completion Time Prediction in a Manufacturing Workshop

**DOI:** 10.3390/s25206480

**Published:** 2025-10-20

**Authors:** Wei Chen, Liping Wang, Changchun Liu, Zequn Zhang, Dunbing Tang

**Affiliations:** College of Mechanical and Electrical Engineering, Nanjing University of Aeronautics and Astronautics, Nanjing 210016, China; chenyichen_412@nuaa.edu.cn (W.C.); liuchangchun@nuaa.edu.cn (C.L.); zhjj370@nuaa.edu.cn (Z.Z.); d.tang@nuaa.edu.cn (D.T.)

**Keywords:** data analysis, feature dataset, deep learning, order remaining completion time prediction, CNN–BiLSTM–Attention

## Abstract

Manufacturing workshops operate in dynamic and complex environments, where multiple orders are processed simultaneously through interdependent stages. This complexity makes it challenging to accurately predict the remaining completion time of ongoing orders. To address this issue, this paper proposes a data-driven prediction approach that analyzes key features extracted from multi-source manufacturing data. The method involves collecting heterogeneous production data, constructing a comprehensive feature dataset, and applying feature analysis to identify critical influencing factors. Furthermore, a deep learning optimization model based on a Convolutional Neural Network (CNN)–Bidirectional Long Short-Term Memory (BiLSTM)–Attention architecture is designed to handle the temporal and structural complexity of workshop data. The model integrates spatial feature extraction, temporal sequence modeling, and adaptive attention-based refinement to improve prediction accuracy. This unified framework enables the model to learn hierarchical representations, focus on salient temporal features, and deliver accurate and robust predictions. The proposed deep learning predictive model is validated on real production data collected from a discrete manufacturing workshop equipped with typical machines. Comparative experiments with other predictive models demonstrate that the CNN–BiLSTM–Attention model outperforms existing approaches in both accuracy and stability for predicting order remaining completion time, offering strong potential for deployment in intelligent production systems.

## 1. Introduction

Order remaining completion time prediction is an essential factor in production process control, affecting the timely delivery of products and reducing product inventory. Early scholars proposed that the product delivery system may not be able to meet any completion date successfully, so the rationality of specifying the remaining completion time directly affects the robustness and stability of the entire manufacturing system [1,2]. The remaining completion date can be measured by the consistency of the processing schedule with the specified due date. Specific metrics are functions of workflow time and specified deadlines, such as working late and working early. Order remaining completion time prediction is a key task in predictive process monitoring, aiming to forecast the completion time of ongoing orders [3]. This task plays a vital role in optimizing system performance, improving resource allocation, and supporting timely decision-making. During the order production progress in the manufacturing workshop, if the remaining completion time of the order can be accurately predicted, the delivery rate of the order can be effectively improved, and it can also provide a decision-making basis for workshop process optimization [4]. Therefore, accurately estimating and shortening the cycle time of every job in the workshop is a prerequisite. Setting accurate delivery dates and ensuring on-time delivery improve customer satisfaction and loyalty. They also enhance communication efficiency and service quality. Moreover, timely delivery provides organizations with competitive advantages in cost efficiency, supply chain synergy, and risk management [5].

In the aerospace industry, discrete manufacturing workshops typically process components such as shafts, plates, and brackets. The production process involves multiple machines performing turning, milling, grinding, and drilling operations. These workshops are characterized by dynamic, order-driven, and multi-machine production environments. The presence of complex process routes, heterogeneous equipment states, and uncertain disturbances poses significant challenges for accurately predicting the remaining completion time of manufacturing orders. When an order arrives on the workshop, it is assigned a deadline indicating when the job is expected to be completed. Each arriving task must be processed on a specific machine in the workshop. As tasks move through the shop floor, different machines experience varying levels of congestion. These fluctuations in workload significantly affect the machining cycle time [6]. The average processing cycle of some complex products may be several weeks or even months, and the processing cycle deviation may be tens or even hundreds of hours. Therefore, many studies have demonstrated the challenge in accurately predicting the remaining completion time of manufacturing systems [7]. There are generally two methods for predicting the cycle time of a job. The first method utilizes artificial intelligence methods (e.g., neural networks [8], deep learning [9], and linear regression [10]). By analyzing and processing certain factors that affect work cycle time, a relationship model between cycle time and these factors is established to predict the order remaining completion time. The second method is to use the time series method to treat the fluctuation of work cycle time as a time series and then analyze the manufacturing process data in time series to predict the remaining completion time of the order [11]. In summary, many methods for predicting the remaining completion time of orders rely on mining and analyzing various data types generated during the manufacturing process. This highlights that the collection, processing, and analysis of manufacturing data play a crucial role in production management. Consequently, data-driven approaches have become essential for improving manufacturing efficiency and accuracy [12]. In addition, with the increasing demand for customer customization, the manufacturing model of modern enterprises has changed from inventory-oriented to order-oriented. Under the challenges of this new manufacturing model, formulating a suitable completion cycle and predicting accurate completion time when abnormal disturbances occur in the production process are crucial to meeting customer needs. Future manufacturing will feature large-scale production, diverse machines, and complex process routes. The workshop environment will be highly dynamic, making cycle time prediction for part batches a task with large-scale prediction targets and intricate constraints [13]. Consequently, it is crucial for manufacturing workshop managers to obtain real-time progress information on scheduled orders to balance the differences between the original production plan and the actual manufacturing process.

Therefore, this paper proposes a method for predicting the remaining completion time of manufacturing workshop orders by combining data-driven approach with CNN–BiLSTM–Attention. The main contributions of this paper are as follows:

(1) Through systematic data preprocessing, key patterns and hidden factors affecting processing cycle fluctuations are effectively identified, while noise reduction and missing value handling enhance data quality for more accurate and reliable analysis.
(2) Utilizing deep learning to build predictive models enables workshop managers to explore production data more thoroughly, thereby uncovering complex relationships between various inputs (e.g., order data, workshop status, and machine conditions) and outputs (predicted remaining completion time).
(3) By integrating CNN, BiLSTM, and Attention mechanism, this paper enhances the model ability to capture spatial patterns, temporal dependencies, and key contextual features in manufacturing processes, thereby achieving more accurate and reliable predictions of order remaining completion time.

The prediction approach proposed is particularly suitable for discrete, order-driven manufacturing industries, such as aerospace, automotive components, and precision machining. These industries are characterized by multi-stage operations, batch processing, and frequent resource reallocation. The approach assumes that manufacturing processes are discrete rather than continuous, and that sufficient historical data can be obtained from workshop information systems. However, this approach may not be directly applicable to continuous or process-oriented industries, where production flows, process dependencies, and temporal dynamics differ significantly. Under these boundary conditions, the proposed method provides a robust and transferable framework for predicting the remaining completion time in discrete manufacturing workshops with complex and dynamic operational characteristics.

The rest of this paper is organized as follows: Section 2 reviews the related works. Section 3 describes the construction process of the feature dataset for predicting the remaining completion time of orders. Section 4 proposes a deep learning optimization model based on CNN–BiLSTM–Attention for the remaining completion time prediction. Section 5 presents the experiments and results. Finally, Section 6 provides the conclusions and directions for future research.

## 2. Related Works

### 2.1. Workshop Remaining Completion Time Prediction

The order completion time is generally specified by the company based on the delivery date. The completion time should be slightly ahead of the delivery date to leave margin for abnormal situations in the production process. Completion time forecasting can generally be divided into three conceptual categories. The first is shop-free load analysis. Order completion time prediction is based on average completion times derived from similar products that have been fulfilled in the past [14]. The second is an analysis based on the total load of the workshop. Completion time is considered based on the overall usage of all resources on the workshop, especially bottleneck resources [15]. The third is based on a detailed analysis of workshop loads. Utilizing real-time information on load status on the manufacturing workshop for order remaining completion time prediction [16]. Although more and more researchers focus on detailed analysis based on workshop load, they still use some variable and ideal production data (e.g., the arrival time of work in process, the processing time of all operations, waiting time of each operation) to represent the detailed workshop load [17]. In recent years, scholars have developed several methods for predicting order completion cycles. Hsu and Sha [18] proposed a due date allocation rule based on artificial neural networks and combined with statistical analysis methods to analyze the production forecast results. Vinod et al. [19] used production simulation to study the interaction between due date allocation methods and scheduling rules in dynamic job shop production systems. Brahimi et al. [20] used mixed integer linear programming methods to study the dependence between workload and lead time. Wang et al. [21] used artificial neural networks combined with classical heuristic rules to predict the remaining order completion time and proposed future research directions that incorporate both static and dynamic characteristics. Choueiri et al. [22] proposed a hybrid forecasting model based on transition systems and statistical regression for forecasting remaining cycle time in industrial environments. Huang et al. [23] proposed a hybrid method that combines machine learning, system model analysis, mathematical models, and recurrent neural networks to achieve product completion time prediction.

With the development of simulation technology, simulation methods have been widely used in remaining completion time prediction. In theory, production simulation is the most accurate method of estimating cycle times if the simulation model is entirely valid and updated continuously. Most studies build simulation models of manufacturing systems to evaluate various input control and scheduling strategies, and cycle times can be easily obtained in the models [24]. As manufacturing systems become larger and larger, it becomes increasingly difficult to build a detailed model. Large simulation systems are running increasingly slowly, which means the time cost of cycle forecasting in large manufacturing systems is high. In large-scale manufacturing systems where disturbances and changes occur randomly, both analytical and simulation methods may lose their applicability. The artificial intelligence method treats the manufacturing system as a black box. Without knowing the structure of the manufacturing system in advance, designing typical input features and prediction models can estimate the processing time of the workpiece. Therefore, using high-quality input data and accurate prediction models is of great significance to improve prediction accuracy.

However, in the improvement of artificial intelligence models for cycle time prediction, most literature focuses on designing more complex neural networks or data mining models to approximate the predicted values. Relatively few studies have considered the potential of selecting highly correlated input data to estimate output values, which is also an important issue in AI-based methods. The manufacturing workshop contains a large number of different types of data during the processing process, and these data hide the operating rules of the workshop. The deep learning model trained using massive data can better fit the actual operating status of the workshop, and the prediction results are closer to the actual situation [25]. Compared with traditional statistical analysis and shallow neural networks, deep learning can extract high-level features from large-scale, low-value density samples; can obtain valuable knowledge; and has stronger generalization capabilities to process big data [26]. Therefore, it is feasible to study the data information of the manufacturing workshop processing process and use deep learning to establish a prediction method for the remaining completion time.

### 2.2. Deep Learning for Remaining Completion Time Prediction

As an important branch in the field of artificial intelligence, deep learning has achieved tremendous development and application in recent years. By constructing a multi-level neural network structure, deep learning model achieves the ability to efficiently learn and recognize complex patterns in large-scale data [27]. Deep learning can learn multi-level, abstract feature representations from raw data, thus achieving breakthrough results in various tasks [28]. In the development process of deep learning, the continuous evolution of neural network structure is a crucial part. From the earliest multi-layer perceptrons to CNN [29], Recurrent Neural Networks (RNN), and LSTM [30], each network structure has demonstrated strong performance in specific fields. Given the massive and high-dimensional data generated in the manufacturing process, deep learning methods can represent the original data in various ways, such as through automatic classification, regression, clustering, and pattern recognition [31]. In practical applications, neural networks can obtain possible results even if the input data is incomplete or noisy. The neural network model produces better forecasting results compared to traditional methods for predicting completion time [32]. Deep learning is compelling in discovering complex structures in high-dimensional data and therefore has enormous applications in the manufacturing field. There is also a growing body of literature on using deep learning for production prediction. Backus et al. [33] used historical data to learn a prediction model of cycle time and used modern data mining algorithms to develop a nonlinear prediction method suitable for most process batches. Chien et al. [34] used a method combining gauss-newton regression and back-propagation neural network to predict semiconductor production line cycle time. Lin et al. [35] developed a hybrid self-supervised/supervised learning framework for remaining useful life prediction, addressing data scarcity and sensor noise using non-full lifecycle data. Liu et al. [36] proposed a channel-spatial attention-based temporal network with channel spatial mechanisms and receptive field optimization for predictive health management performance. Liu et al. [37] introduced an LSTM–self-attention hybrid multi-target regression framework for accurate order completion time prediction in dynamic make-to-order production.

With the development of smart manufacturing, more and more machines in manufacturing workshops are equipped with intelligent sensors. However, most manufacturing companies also lack the software and models to interpret and analyze this data. As manufacturing processes become more complex, data analysis and modeling become increasingly difficult. Deep learning can automatically mine knowledge from aggregated data and plays a crucial role in data prediction and production decision-making. Ding and Jiang [38] proposed an RFID-based production data analysis method, aiming to use the production data of the IoT intelligent job shop to improve and optimize production predictions. Fang et al. [39] proposed a stacked sparse autoencoder-based model utilizing production big data, which achieved accurate prediction of job remaining time. Liu et al. [40] proposed a stacking denoising auto-encoder with a sample weight method, combining transfer learning and feature extraction technology to predict order remaining completion time. Due to the advancements in deep learning, LSTM networks have been widely used in the field of manufacturing process prediction due to their unique gating structure design, which performs well in handling temporal correlation and nonlinearity of data. Yuan et al. [41] proposed an optimization method based on the Auto-encoder-CNN-LSTM model to analyze production data to predict order completion time. Liu et al. [42] proposed a production progress prediction method using a transfer learning model, combining a convolutional neural network and an LSTM network to mine the comprehensive characteristics of historical and current orders to improve prediction performance. Wang et al. [43] proposed a real-time extensive data processing method for the workshop production process based on LSTM, utilizing these models to train and predict data from the IoT job shop. However, the LSTM network can only learn forward temporal features. The BiLSTM is an extension of the LSTM neural network, which is characterized by understanding the bi-directional timing characteristics of the data, fully considering historical and future information, and further improving the prediction.

The above references mainly focus on point forecasting, which is difficult to meet in the fine-tuned management of manufacturing workshop. In a multi-disturbance and uncertain manufacturing workshop environment, changes in production tasks, machine performance, and technical requirements lead to changes in the correlation between prediction targets and manufacturing data, and the accuracy of fixed prediction models can decrease or even be invalidated. Through the intensive study of deep learning, completion time prediction mainly involves two aspects. First, a feature selection method is applied to analyze the correlation between potential influencing factors of the remaining order completion time, resulting in a dataset composed of key features. Second, a deep learning model is constructed, where the subset of key features is used as input. The data is propagated forward, and the activation function determines the network output at each layer, yielding the target prediction value. Then, the prediction error is back-propagated, and a suitable optimization algorithm is selected based on the loss function to adjust the network parameters. The training is iteratively updated until a high-precision prediction model is obtained, capable of fitting the operating rules of the workshop. Finally, the real-time data corresponding to the workshop is input into the model to predict the remaining completion time of the order, determine whether the order can be completed on time, and realize the application value of manufacturing data.

### 2.3. Research Gaps and Methodology

Based on existing studies on order remaining completion time predictions in manufacturing workshops, significant progress has been made in deep learning methods. However, critical research gaps remain unresolved, thus limiting the accuracy and adaptability of current approaches in dynamic production environments.

(1) Although deep learning models have been widely adopted, most studies have focused on isolated temporal or spatial feature extraction. For instance, traditional LSTM networks excel in capturing sequential dependencies but neglect local spatial patterns in manufacturing data. Conversely, CNN-based methods prioritize spatial correlations but fail to model long-term temporal dependencies, which leads to suboptimal predictions under complex multi-factor interactions.
(2) The current approaches predominantly target static or idealized production conditions by relying on fixed input features and deterministic predictions. In real-world workshops with frequent disturbances, the correlation between input features and prediction targets shifts dynamically, which causes model performance degradation. Existing methods lack adaptive mechanisms to update feature relevance or adjust model structures in real time.
(3) Although manufacturing systems generate high-dimensional heterogeneous data, most studies either adopt a simplistic feature selection or directly feed raw data into complex networks without optimizing the information density. This results in redundant computations and poor interpretability, particularly when handling sparse or noisy datasets.

To address the aforementioned research gaps, this paper follows a data-driven research methodology consisting of four main stages. The overall process aims to establish an accurate and adaptive prediction model for the remaining completion time of manufacturing orders under dynamic production environments.

(1) Data collection and preprocessing. Using data collection methods such as RFID, PLC, and 5G, structured production data is extracted from multiple manufacturing information systems through extract, transform, and load technology. Then, original feature dataset is constructed by orders, work in process, and machine-level data. To ensure data quality, perform preprocessing on the dataset, including outlier handling, missing value imputation, and data normalization. This preprocessing stage ensures that the dataset is both representative and suitable for deep learning model training.
(2) Model design and implementation. To overcome the limitations of existing single-structure models, this paper develops a hybrid CNN–BiLSTM–Attention deep learning model. The CNN component is used to extract local spatial features from high-dimensional production data, while the BiLSTM layer captures bidirectional temporal dependencies to model sequential production patterns. The integrated Attention mechanism adaptively assigns weights to key features and time steps, enabling the model to emphasize critical information and reduce noise influence. This hybrid architecture facilitates comprehensive spatiotemporal feature learning for accurate order completion time prediction.
(3) Experimental setup and validation. Experimental studies are conducted using real workshop data collected from a production line equipped with ten types of machining equipment. Multiple comparative methods, including Back Propagation (BP), Deep Belief Network (DBN), Convolutional Neural Network (CNN), Bidirectional Long Short-Term Memory (BiLSTM), CNN-LSTM and CNN-BiLSTM, are implemented to benchmark performance. Evaluation metrics such as mean absolute error, root mean square error, and R2 are employed to quantify prediction accuracy and generalization ability. Sensitivity and robustness analyses are also performed to verify the model’s adaptability under dynamic production conditions.
(4) Result interpretation and analysis. Finally, the predictive performance and interpretability of the proposed model are analyzed through visual comparisons and statistical evaluations. The results demonstrate that the proposed CNN–BiLSTM–Attention model effectively captures spatiotemporal dependencies and outperforms baseline models in both accuracy and robustness, providing strong methodological support for data-driven decision-making in discrete manufacturing workshops.

## 3. Feature Dataset Modeling

### 3.1. Problem Constraints

When predicting the remaining completion time of workshop orders, the following constraints should be considered to ensure comprehensive and realistic modeling.

(1) Resources constraint: Resources in discrete manufacturing workshops, including machines, human labor, and materials, play a crucial role in determining production efficiency. Their availability, allocation, and utilization directly influence both production performance and schedule feasibility. Material constraints often arise from shortages or delays in the supply of raw materials or semi-finished components. To address these issues, accurate procurement planning, dynamic inventory management, and real-time monitoring of material consumption are required to maintain the balance between supply and demand. Equipment constraints mainly stem from machine reliability issues and maintenance requirements. Unplanned downtime caused by equipment failures, as well as scheduled calibration or repairs, can disrupt production.
  To mitigate these risks, predictive maintenance strategies and the preparation of backup equipment should be integrated into the production system. Human resource constraints are reflected in limited labor availability, varying skill proficiency, and fluctuating operational efficiency. These challenges call for systematic solutions such as scientific shift scheduling, skill-based training programs, and workload balancing mechanisms to alleviate production bottlenecks. Together, these three dimensions, including materials, equipment, and human resources, form interdependent elements within the resource constraint framework. A systematic optimization approach is therefore required to achieve a dynamic balance between resource utilization efficiency and production rhythm.

(2) Process and operational constraints: In discrete manufacturing workshops, a number of process and operational constraints inherently influence the execution of production tasks. Different products often follow distinct process routes and machining steps, and certain operations must be executed in a predefined order to ensure the accuracy and continuity of manufacturing processes. Each machine is capable of processing only one operation at a time, while the operations of each part must strictly comply with the process sequence specified in the production plan. In addition, the processing order of parts within in-buffer and out-buffer areas generally follows a first-in-first-out (FIFO) rule to maintain workflow stability and production efficiency.
  Violations of these process sequences may lead to machine idle time or production delays. Therefore, effective process modeling should explicitly account for task precedence rules, inter-task dependencies, and machine exclusivity constraints to ensure the feasibility and reliability of production schedules. These constraints are inherently embedded in the collected production data and are therefore implicitly reflected in the feature modeling stage. Consequently, the constructed feature dataset inherently captures such production logic, allowing the proposed CNN–BiLSTM–Attention model to learn temporal dependencies and operational behaviors under realistic manufacturing conditions.

### 3.2. Feature Dataset Construction

This section describes the data classification model for predicting order remaining completion time, including the feature selection process and the definition of the objective function. To achieve this, assumptions are made regarding the factors of Work-In-Progress (WIP) status, equipment status, and order status along the processing route to form a predictive data feature set [44]. The status of the WIP includes dimensions such as entry and exit buffer zone time, transportation time, processing time, waiting time, WIP quality, and buffer zone exit time. Key features that affect the final completion time are selected. Equipment status includes several factors (e.g., normal operation, failure, shutdown, and equipment utilization). The order status includes several factors, such as the waiting time before the order reaches the workshop, the circulation time of the order in the workshop, and the processing time of the order in the workshop. The principle of predicting order remaining completion time is to use as few critical features as possible to replace the features of all data and improve the prediction accuracy and speed [45]. Therefore, Table 1 shows all states before, during, and after processing are analyzed to obtain all features that may be related to the completion time, thereby forming a remaining completion time prediction feature dataset.

The entire business process of the manufacturing workshop includes order issuance, order waiting, order processing and other influencing factors. Order release time refers to the delivery of materials from the warehouse to the workshop. The order waiting time refers to the batch to be processed waiting in the queue area for idle machines to arrange processing. The processing process refers to the parts being processed according to the process route. Other influencing factors are some uncontrollable factors in the manufacturing process, such as emergency orders, material problems, equipment failures, etc. Therefore, the overall machining process formula is defined as follows:

(1) $$F(t)=Transport_{ij}+Wait_{ij}+Process_{ij}+Add_{ij}$$

where *i* is the part processing step, *j* is the batch of processed parts.

**Figure 1:**
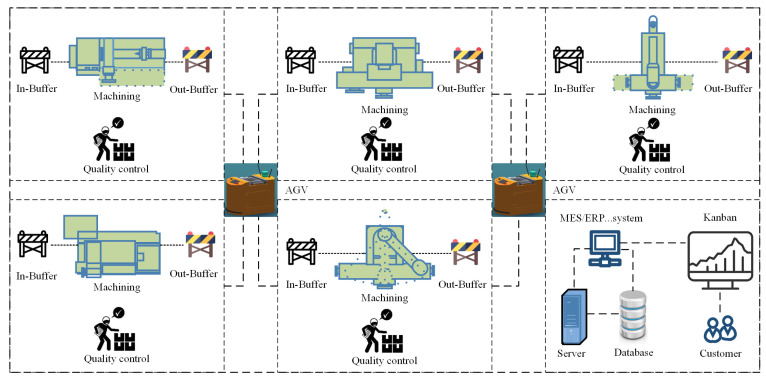
Workshop manufacturing process.

In addition, information systems such as Enterprise Resource Planning (ERP), Manufacturing Execution System (MES), and Warehouse Management System (WMS), together with IoT devices, record all operational data generated throughout the manufacturing process. These systems track the flow of operations between workstations through interconnected business processes. As illustrated in Figure 1, the workshop consists of multiple machining units, each equipped with in-buffer and out-buffer areas for material storage and quality inspection stations for product validation. The Automated Guided Vehicles (AGVs), represented by the orange icons, are responsible for transporting semi-finished products between machining units according to dynamic scheduling instructions issued by the MES. The dashed lines indicate the logistic routes and communication pathways that connect each machining unit to the AGV system, enabling real-time material transfer and task coordination. Data collected from machines, sensors, and AGV controllers are transmitted to the central server and database via a wireless industrial network (e.g., Wi-Fi or 5G-based IoT communication). These data streams are then integrated into the MES/ERP system for monitoring and performance analysis. Furthermore, the influencing factors considered by the prediction model include in-process operations, process routes, product types, quantities of work-in-progress items, product quality status, and equipment conditions.

The production status of the workshop can be composed of WIP status, equipment status MS and order status OS. The WIP includes the status IB of the WIP entering the buffer, the processing status PS, the status OB of the WIP leaving the buffer, the transfer status WS of the WIP when it leaves the buffer area and is transferred to the following processing unit, and the quality status WQ of the WIP. The above three types of information on WIP status, equipment status, and order status are categorized as the state set at a particular moment. Then, each set of interconnected IB, PS, OB, WS, WQ, MS, and OS constitutes a complete set of production state features at a particular moment in the workshop. Therefore, the state of the manufacturing workshop that affects the order remaining completion time prediction can be defined as follows.

The definition of WIP status refers to the status of products that have not yet been completed in the production process, including waiting for production, in production, production suspended, quality control, on hold, and production completed six statuses.

Equipment status definition means a term or classification used to describe the operational condition or state of equipment. These states help monitor equipment operation, maintain equipment, schedule tasks, and predict possible failures, which include idle, running, paused, fault, maintenance, shut down, ready, calibration, and awaiting repair in nine states. In order to simplify the calculation, this paper only adopts the four states of running, idle, maintenance, and shut down to represent the features of the machine.

Order status definitions are the states used to describe the different stages of order processing. These states can help monitor the processing of orders, track the progress of orders, and provide appropriate feedback to the shop floor managers, including pending, processing, confirmed, picked, shipped, completed, canceled, and exception eight states.

Typically, raw datasets are collected and stored in a distributed database in the workshop information system, including both real-time collected data and historical data covering information such as work-in-progress status, machine status, and order status. The workshop production process is assumed to be processed according to the first in first out scheduling rule. Then, the features of the remaining completion time prediction are formalized as follows:

(2) $$F(t)=\left.\left\{IB_{t},PS_{t},OB_{t},WS_{t},WQ_{t},MS_{t},OS_{t}\right.\right\}$$

The features are formalized as described in detail below.

(1) WIP status

The state of the WIP in the cache is $IB_{t}=\left\{\left.IB_{t}^{d}\left|d=1,2,..,D\right.\right\}\right.$, where $IB_{t}^{d}$ denotes the state of the WIP in the machine *d* at time *t*. The WIP waiting to be processed is stored in an arranged sequence in the buffer. Thus, $IB_{t}^{d}$ can be composed of the WIP type $IT_{t,i}^{d}(IT_{t,i}^{d}\in \{1,2,\ldots ,K\})$ and the waiting time $IW_{t,i}^{d}$ of the *i*-th location of the machine *d* into the cache at time *t*. The formula for entering WIP into the buffer is as follows:

(3) $$IB_{t}^{d}=\left\{\left(IT_{t,i}^{d},IW_{t,i}^{d}\right)\right.\left|i=1,2,\ldots ,\right.\left.I_{d}\right\}$$

where $I_{d}$ represents the capacity of the machine *d* into the buffer capacity, the value is a constant.

The status of the WIP being processed is $PS_{t}=\{PS_{t}^{d}\left|d=1,2,\ldots ,D\right.\}$, and $PS_{t}^{d}$ represents the processing status of machine *d* at time *t*. The WIP processing status consists of the currently processed WIP type $PT_{t}^{d}$ and processing time $WT_{t}^{d}$. The processing status is shown in Equation (4):

(4) $$PS_{t}^{d}=\{PT_{t}^{d},WT_{t}^{d}\}$$

The status of the WIP exit buffer area is $OB_{t}=\left\{\left.OB_{t}^{d}\left|d=1,2,..,D\right.\right\}\right.$, and $OB_{t}^{d}$ represents the exit buffer area status of machine *d* at time *t*. Similarly, it consists of the WIP category $PT_{t,i}^{d}(PT_{t,i}^{d}\in \{1,2,..,K\})$ and the waiting time $OW_{t,i}^{d}$ at the *i*-th location of the machine *d* out of the buffer at time *t*. The formula is expressed as follows:

(5) $$OB_{t}^{d}=\{(PT_{t,i}^{d},OW_{t,i}^{d})\left|i=1,2,\ldots ,O_{d}\right.\}$$

where $O_{d}$ represents the capacity of the machine *d* out the buffer capacity, the value is a constant.

The transfer status of the WIP is $WS_{t}=\{WS_{t}^{d}\left|d=1,2,\ldots ,D\right.\}$.where $WS_{t}^{d}$ denotes the number of *k*-th type of WIP $QP_{t,k}^{d}(QP_{t,k}^{d}\in \{1,2,\ldots ,Q_{k}\})$ and the transfer time $TT_{t,k}^{d}$ constituting the number of WIP coming out of the buffer area of the machine *d* and still in the process of transferring at time *t*. The state representation is shown as follows:

(6) $$WS_{t}^{d}=\{(QP_{t,k}^{d},TT_{t,k}^{d})\left|k=1,2,\ldots ,K\right.\}$$

The quality status of the work in progress is $WQ_{t}=\{WQ_{t}^{q}|q=1,2,\ldots ,Q\}$. $WQ_{t}^{q}$ consists of the quality inspection quantity $QI_{t}^{q}$ in units of *q* at time *t* and the quality inspection status $QR^{q}$. The state representation is shown as follows:

(7) $$WQ_{t}^{q}=\{(QI_{t}^{q},QR^{q})\left|q=1,2,..,Q\right.\}$$

where $QR^{q}$ represents the four states of qualified, reworked, qualified after rework, and scrap of the WIP, which are indicated by 0, 1, 2, and 3, respectively.

(2) Machine status

The state of the machine is one of the uncertainties that affects the production progress, a machine can only process one WIP at the same time and the operating state of the machine is unique. In this paper, the utilization, real-time status, and the working time of each machine are considered factors that influence the remaining completion time prediction. Assuming that there are *d* machines in the manufacturing workshop, the machine state $MS_{t}^{d}$ at the time *t* can be expressed as follows:

(8) $$MS_{t}^{d}=(DS_{t}^{d},AU^{d},CT_{t}^{d})$$

where $DS_{t}^{d}$ represents the four states of machine running, idle, maintenance, and shutdown, which are represented by 0, 1, 2, and 3, respectively. $AU^{d}$ represents the average usage rate of machine tools from time *t* − 1 to *t*. $CT_{t}^{d}$ represents the continuous working time of the machine since the last maintenance.

(3) Order status

Multiple production orders in the manufacturing workshop will be processed simultaneously on different equipment according to different process routes. In this paper, production tasks are used to represent the quantity of production orders that need to be processed in the workshop. Generally, the types of parts that need to be made in the shop are broadly fixed, and the variation between orders is the number of different part types. Therefore, the entire workshop order can be made up of the number of shop orders $OQ_{t}^{k}$ and the number of parts $PQ_{t}^{n}$ for each order. The orders $OS_{t}=\{OS_{t}^{q}|q=1,2,\ldots ,Q\}$ to be processed by the workshop are used to influence the prediction of the remaining completion time in this paper, and the order formula is expressed as follows:

(9) $$OS_{t}^{q}=(OQ_{t}^{k},PQ_{t}^{n}\left|k=1,2,\ldots ,K,n=1,2,\ldots ,N\right.)$$

Therefore, the feature dataset F(t) used for order remaining completion time prediction can be expressed as follows:

(10) $$\begin{array}{ll} F(t) & =\left.\left\{IB_{t},PS_{t},OB_{t},WS_{t},WQ_{t},MS_{t},OS_{t}\right.\right\}\\ & =\left\{\begin{array}{l} IT_{t,i}^{d},IW_{t,i}^{d},PT_{t}^{d},WT_{t}^{d},PT_{t,i}^{d},\\ OW_{t,i}^{d},QP_{t,k}^{d},TT_{t,k}^{d},QI_{t}^{q},QR^{q},\\ DS_{t}^{d},AU^{d},CT_{t}^{d},OQ_{t}^{k},PQ_{t}^{n} \end{array}\right\},\\ & \;(d=1,\ldots ,D,q=1,\ldots ,Q,k=1,\ldots ,K,n=1,\ldots ,N) \end{array}$$

### 3.3. Data Preprocessing

**Figure 2:**
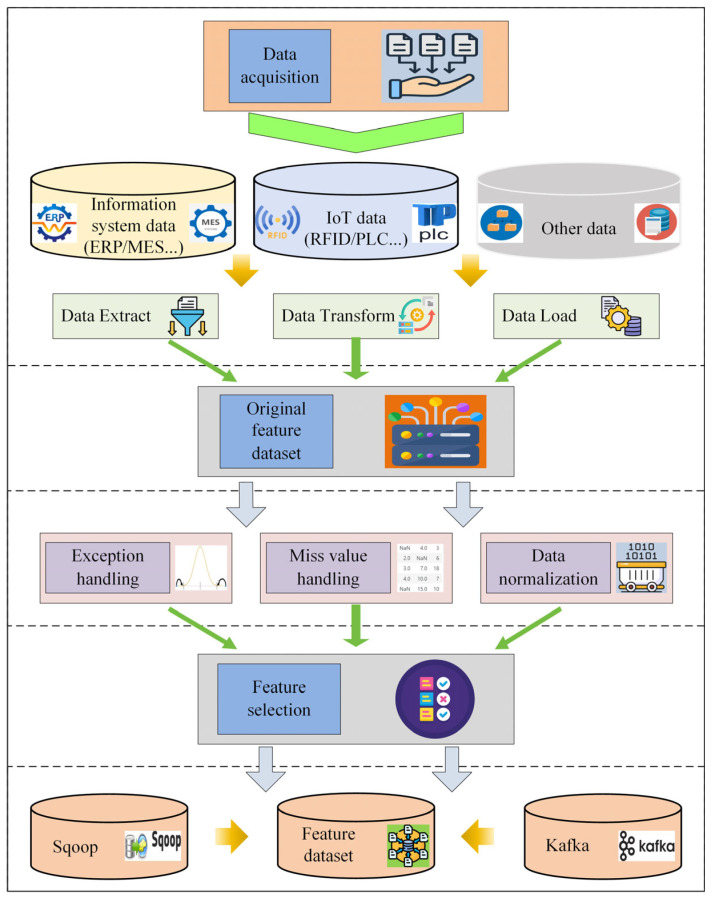
Data preprocessing flow.

The sources of manufacturing workshop data mainly include data obtained from information systems, IoT data, and external data. Data from information systems refers to the data generated and stored in ERP, MES, WMS, and other related systems. Data from IoT refers to the data obtained through RFID, PLC, and 5G, etc. External data refers to data originating from customers or suppliers. These candidate data are characterized by massive volume, high dimensionality, and heterogeneity. Regarding the volume of data, the prediction candidate dataset for hundreds or thousands of orders comes to hundreds of thousands or even millions of entries. In terms of the dimensions of the data, the candidate dataset contains various types of data on WIP, multiple types of machine status data, and multiple types of order data. In terms of the structure of the data, the candidate data covers a wide range of data structure types, such as linear, nonlinear, and temporal types. These multiple sources of heterogeneous, high-dimensional, massive data augment the challenges of data analysis in order remaining completion time prediction. Therefore, Figure 2 analyzes the data preprocessing workflow from several dimensions: data acquisition, construction of the raw feature dataset, data cleaning, feature selection, and data integration.

First, data acquisition mainly ensures that all types of data in the manufacturing process can be stored in the database quickly and stably. For different types of data, different methods are used for data acquisition. Structured data is collected from ERP, MES, WMS, and other information systems using Extract, Transform, Load (ETL) technology. Large-volume data is extracted using ETL technology, while small-volume data is collected through API interfaces. For real-time data generated from devices such as RFID, PLC, or 5G, Kafka is suitable for data collection.

Then, the result of original feature dataset contains a large amount of timing data, including processing time, waiting time, and other key timing features. There are different scales between these data, which need to be standardized to speed up model convergence and improve model stability and generalization. Due to the discrete and random properties of the production process, there may be many incomplete, inaccurate, and repetitive data. Therefore, it is necessary to handle outliers and missing values in the data and perform data normalization.

Abnormal values may arise for various reasons, such as production stoppages, personnel departures, system anomalies, or other special circumstances during a certain period. These values are typically characterized by significant deviations from normal, including unusually large errors or even zero values. If the abnormal values are not eliminated, the stability of the data and the accuracy of the prediction results will be seriously affected in the prediction process. Therefore, this paper sets a standard range for each type of characteristic data based on production requirements and applies the capping method to handle abnormal data, thereby making the data distribution more reasonable. The upper and lower values of the feature dataset are first set (as shown in Equation (11)), and then the anomalies in the dataset are analyzed and replaced (as shown in Equation (12)).

(11) $$\left\{\begin{array}{l} {f(x)}_{lower}=f(m)-k\times IQR\\ {f(x)}_{upper}=f(n)+k\times IQR \end{array}\right.$$

(12) $$f(x_{i})=\left\{\begin{array}{l} {f(x)}_{lower},\;if\;f(x_{i})<{f(x)}_{lower}\\ {f(x)}_{upper},\;if\;f(x_{i})>{f(x)}_{lower}\\ f(x_{i}),\;\;\;otherwise \end{array}\right.$$

where f(m) is the quarter-bit data in the feature dataset and f(n) is the three-quarter-bit data in the feature dataset, IQR=f(n)−f(m), k is a constant taking the value 3.

Missing values are a common issue in data preprocessing. The reasons for these missing values can include various scenarios, such as data not being captured due to network issues, loss of data from system malfunctions, or equipment failures preventing data collection. The handling of missing values typically involves both deletion and imputation. The research in this paper is mainly oriented to industrial big data in manufacturing workshop, with extensive data volume and high dimensionality. To ensure the accuracy of the prediction results, the missing values are filled in using median interpolation. The median is insensitive to missing values and is more robust when dealing with missing data. The median is the value in the middle of a set of data in sequential order, or the average of the two middle values. For missing values in a feature class, replace them with the median of the non-missing values in that class of feature data, as shown in Equation (13).

(13) $$\tilde{f(x)}=\left\{\begin{array}{l} {f(x)}_{\frac{m+1}{2}},m\;is\;odd\;number\\ \frac{f{\left(x\right)}_{\frac{m}{2}}+f{\left(x\right)}_{\frac{m}{2}+1}}{2},m\;is\;even\;number \end{array}\right.$$

where m is the number of non-missing values and $f{\left(x\right)}_{m}$ is the non-missing value feature data.

Second, the original feature dataset is formed by preprocessing the collected data for missing values and outliers. As the processed feature dataset has 1116 features (further discussed in experimental section) with high feature dimensions, further analysis of the feature dataset is required. Regularization is an efficient and interpretable method for determining feature weights, which is particularly suitable for feature selection in high-dimensional data. By introducing regularization constraints, the model automatically filters out key features and quantify their importance during the fitting the process. The objective function as shown in Equation (14).

(14) $$\underset{\mu }{\min}(\frac{1}{2N}\sum_{i=1}^{N}{\left(f_{i}(x)-\mu_{0}-\sum_{j=1}^{p}x_{ij}\mu_{j}\right)}^{2}+\tau \sum_{j=1}^{p}\left|\mu_{j}\right|)$$

where N is the number of samples, $f_{i}(x)$ is the value of the target variable for the *i*-th sample (i.e., the remaining completion time of the order), $\mu_{0}$ is the model intercept term indicating the baseline predicted value when all features are zero, p is the number of features, $x_{ij}$ is the value of the *j*-th feature variable for the *i*-th sample, τ is the regularization strength parameter (controlling for complexity and sparsity in the model), and $\mu_{j}$ is the regression coefficient for the *j*-th feature (i.e., the weight for the feature).

The coefficients of irrelevant features are compressed to zero by L1 regularization and the desired features are selected. The absolute value of the non-zero coefficient $\left|\mu_{j}\right|$ represents the quantitative value of the feature weights, reflecting the strength of the feature contribution to the remaining completion time prediction. The optimal τ is selected by K-fold cross-validation, thus reducing the prediction error of the feature dataset. Next, the feature weights are computed. Under the optimal parameter τ, the features of $\mu_{j}\neq 0$ are retained to extract the weights, and the absolute values of these weights are subsequently normalized using min-max normalization to scale them within the range [0, 1], as shown in Equation (15).

(15) $$\varphi_{j}=\frac{\left|\mu_{j}\right|-\min(\left|\mu \right|)}{\max(\left|\mu \right|)-\min(\left|\mu \right|)}$$

**Figure 3:**
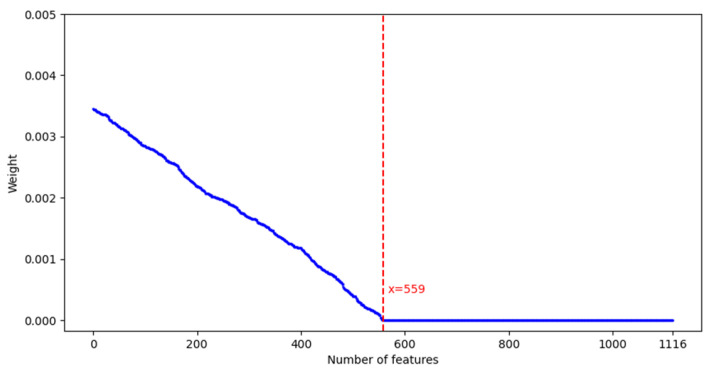
Feature selection for optimal feature dataset.

The features of $\varphi_{j}\geq 0$ are retained and finally the 558 valid features most relevant to predicting the remaining completion time are retained to construct the final feature dataset (as shown in Figure 3).

The final feature dataset is also normalized. Data normalization refers to standardizing data of different types and dimensions generated during the manufacturing process, with the range fixed between [0, 1]. Due to the varying dimensions and different orders of magnitude of the collected data, if the data are used directly without normalization, it is impossible to analyze the prediction results accurately. There are many ways to normalize the data. In this paper, the maximum-minimum normalization method is employed, as shown in Equation (16).

(16) $$f_{i-normal}=\frac{f_{i}(x)-f_{\min}(x)}{f_{\max}(x)-f_{\min}(x)}$$

where $f_{i-normal}$ denotes the normalized data of feature data $f_{i}(x)$, $f_{\max}(x)$ denotes the maximum value of feature data $f_{i}(x)$, and $f_{\min}(x)$ denotes the minimum value of feature data $f_{i}(x)$.

Subsequent to the introduction of the normalized feature data into the prediction model, the output predicted values of the remaining completion time must undergo an inverse normalization operation to complete the recovery of the data, as shown in Equation (17).

(17) $$f(x)=f_{i-normal}\times (f_{\max}(x)-f_{\min}(x))+f_{\min}(x)$$

Finally, data integration refers to the correlation of processed data according to actual requirements. It primarily employs integration methods, including Sqoop and Kafka, to fuse data into feature dataset.

## 4. Optimization Algorithm for Order Remaining Completion Time Prediction

Discrete manufacturing workshops often face significant challenges in managing high-dimensional, nonlinear, and dynamically evolving data. These challenges are generated from complex operations, equipment variability, process uncertainties, and unforeseen disturbances. Accurate and timely prediction of the remaining processing time for manufacturing orders is crucial for effective scheduling, resource allocation, and proactive management of potential delays.

**Figure 4:**
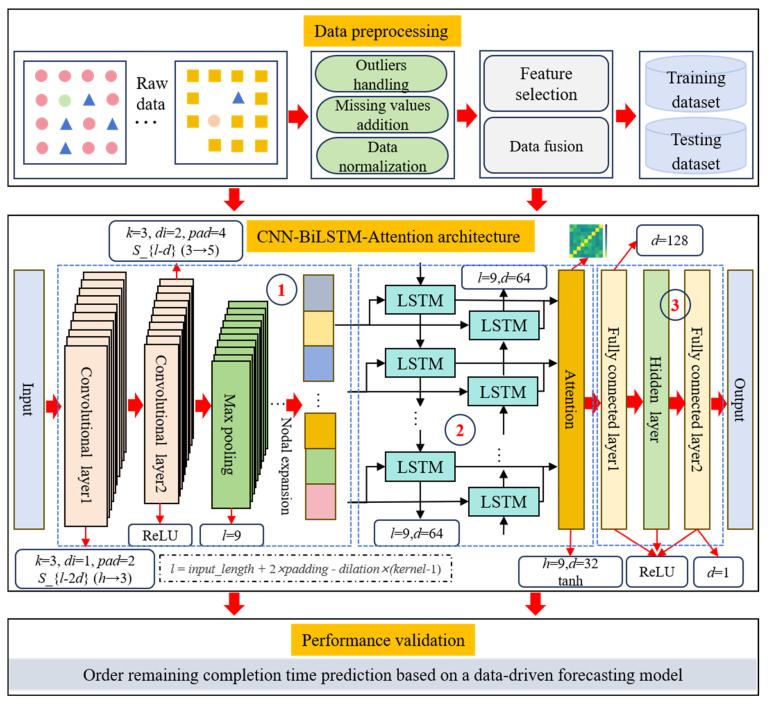
CNN–BiLSTM–Attention-based frameworks for order remaining completion time prediction.

To address these practical engineering challenges and the unique characteristics of manufacturing data, this paper proposes an optimized deep learning approach. The model leverages a CNN–BiLSTM–Attention architecture specifically designed for predicting the remaining completion time of manufacturing orders. As illustrated in Figure 4, the overall framework consists of three interconnected layers. The first layer is responsible for data preprocessing, which includes outlier handling, missing value imputation, and data normalization. It is followed by feature selection and data fusion to construct representative training and testing datasets. The second layer implements the core CNN–BiLSTM–Attention architecture, which comprises three tightly integrated modules: (1) multi-dimensional feature extraction through a multi-kernel CNN to capture hierarchical spatial correlations from heterogeneous workshop data, (2) context-aware attention-based key feature refinement via a BiLSTM–Attention block that adaptively identifies critical temporal dependencies influencing completion times, and (3) high-precision prediction achieved through a nonlinear-linear fusion mechanism that transforms complex temporal-spatial relationships into interpretable scalar outputs. The third layer conducts model performance validation using evaluation metrics to quantitatively assess prediction accuracy and generalization capability, thereby ensuring the reliability of the proposed forecasting model.

(1) Multi-dimensional feature extraction module

Multi-dimensional feature extraction module introduces a multi-kernel CNN-based feature extractor, which performs parallel convolution operations across multiple temporal and spatial axes of the manufacturing dataset. Unlike traditional handcrafted feature engineering or shallow networks, the proposed design leverages deep convolutional operations to automatically capture hierarchical spatial patterns embedded in heterogeneous workshop data, such as machine status, operation queues, and WIP attributes.

The convolutional component transforms raw multivariate time-series data into high-level feature maps while preserving local correlations and suppressing redundancy through max-pooling. The convolution operation is formulated in Equation (18), which defines how local contextual information is aggregated under different receptive fields. This hierarchical feature abstraction ensures that subsequent layers are fed with noise-reduced, information-dense representations.

(18) $$F_{k}^{l}(t)=f(\sum_{c=1}^{C_{l-1}}\sum_{i=0}^{w-1}W_{k,c,i}^{l}X_{c}^{l-1}(t+i)+b_{k}^{l})$$

where $W_{k,c,i}^{l}$ represents the weight of the *k*-th convolution kernel at layer *l*, associated with channel *c* and offset *i*, $X_{c}^{l-1}(t+i)$ represents the feature value at time step *t* + *i* of the *c*-th channel in the (*l* − 1)-th layer, $b_{k}^{l}$ represents the bias parameter associated with the *k*-th convolutional kernel in the *l*-th layer.

(2) Attention-based key feature refinement module

Attention-based key feature refinement module introduces temporal sequence modeling and adaptive weighting through a BiLSTM–Attention hybrid block. On the one hand, the BiLSTM layer captures long-range bidirectional dependencies in production event sequences, enhances the model ability to capture complex, nonlinear relationships over time, and addresses the temporal complexity in predicting the remaining completion time of an order. The equations are shown as follows:

(19) $${\vec{h}}_{t}=LSTM_{fwd}(x_{t},{\vec{h}}_{t-1})$$

(20) $${\overset{\leftarrow }{h}}_{t}=LSTM_{bwd}(x_{t},{\overset{\leftarrow }{h}}_{t+1})$$

The core computation formulas for each LSTM unit at a single time step (taking the forward direction as an example) are as follows:

(21) $$i_{t}=\delta (W_{i}(x_{t},{\vec{h}}_{t-1})+b_{i})$$

(22) $$f_{t}=\delta (W_{f}(x_{t},{\vec{h}}_{t-1})+b_{f})$$

(23) $$o_{t}=\delta (W_{o}(x_{t},{\vec{h}}_{t-1})+b_{o})$$

(24) $${\tilde{c}}_{t}=\tanh(W_{c}(x_{t},{\vec{h}}_{t-1})+b_{c})$$

(25) $$c_{t}=f_{t}⊙c_{t-1}+i_{t}⊙{\tilde{c}}_{t}$$

(26) $${\vec{h}}_{t}=o_{t}⊙\tanh c_{t}$$

The concatenation of the bidirectional hidden states at each time step is shown as follows:

(27) $$h_{t}=[{\vec{h}}_{t}||{\overset{\leftarrow }{h}}_{t}]$$

where the variables $i_{t}$, $f_{t}$, and $o_{t}$ represent the activation values of the input gate, forget gate, and output gate, respectively, each constrained within the range [0, 1]. $W_{i}$, $W_{f}$, $W_{o}$, $W_{c}$ correspond to the weight matrices of the input gate i, forget gate f, output gate o, and cell gate $\tilde{c}$, respectively. $b_{i}$, $b_{f}$, $b_{o}$, and $b_{c}$ correspond to the bias vectors of the i, f, o, and $\tilde{c}$, respectively. $c_{t}$ represents the cell state of the LSTM, which carries long-term memory, while ${\tilde{c}}_{t}$ represents the candidate cell state.

On the other hand, the incorporation of the attention mechanism significantly enhances the model’s ability to dynamically identify critical features. In real-world manufacturing environments, rare but impactful events (e.g., equipment failures, irregular production rhythms, or raw material delays) can substantially affect order completion times, yet are often difficult to capture using conventional methods due to their low frequency. The Attention mechanism assigns dynamic weights to the output sequence of the BiLSTM layer, enabling the model to selectively focus on the most informative parts of the input while suppressing noise and irrelevant patterns. This integration not only improves the robustness of the model but also enhances its interpretability, allowing it to more accurately highlight decisive features or events in complex, multi-factor manufacturing systems. The equations are shown as follows:

(28) $$e_{t}=v^{Τ}\tanh(W_{h}h_{t}+b_{h})$$

(29) $$\alpha_{t}=\frac{\exp(e_{t})}{\sum_{k=1}^{T}\exp(e_{k})}$$

(30) $$c=\sum_{t=1}^{T}\alpha_{t}h_{t}$$

where $e_{t}$ represents the unnormalized alignment score, v represents the score vector in the attention mechanism, $W_{h}$ represents the linear transformation matrix, $h_{t}$ represents the concatenated hidden state of the BiLSTM at time step *t*, and $b_{h}$ represents the bias vector used during attention scoring. $\alpha_{t}$ represents the normalized attention weights, c represents the context vector obtained by the weighted sum of the hidden states across all time steps.

Statistical analysis of the trained attention weights is conducted to identify key features that significantly contribute to the prediction of order remaining completion time. These features consistently exhibited high attention scores across different production scenarios, indicating strong predictive relevance. A stable feature set is initially selected using regularization during the data preprocessing phase, while the Attention mechanism subsequently revealed the dynamic contribution patterns of these features during prediction. This combined strategy of static feature selection and dynamic importance evaluation enables a more comprehensive analysis that integrates both feature stability and context-dependent adaptability.

(3) High-precision linear prediction module

The high-precision linear prediction module performs nonlinear–linear fusion to enhance prediction accuracy. It transforms the attention-refined BiLSTM representations into a scalar output that precisely estimates the remaining completion time. This module consists of fully connected layer and hidden layer that first performs nonlinear transformation, followed by a linear output mapping.

Specifically, the attention-weighted context vector c is fed into a dense layer with a ReLU activation function to model complex, nonlinear interactions among the extracted features. This step enables the model to capture nonlinear dependencies and conditional combinations of multiple influencing factors (as shown in Equation (31)).

(31) $$z=\delta (W_{1}c+b_{1})$$

where $W_{1}$ is the weight matrix, $b_{1}$ is the bias term, and δ(⋅) is a nonlinear activation function. The output vector z serves as a compressed and abstracted representation capturing higher-order correlations in the feature space.

Subsequently, this intermediate representation z is passed through a final linear projection layer to generate a scalar output $y^{p}$, which corresponds to the predicted remaining completion time (as shown in Equation (32)).

(32) $$y^{p}=W_{2}z+b_{2}$$

Unlike black-box regressors, this structure is designed to preserve feature separability learned by the attention mechanism, while still enabling linear interpretability of the final output layer. Moreover, this design choice improves convergence during training and facilitates integration with real-time edge devices due to its lightweight output logic, which also allows the model to be efficiently retrained or fine-tuned with new production data using incremental learning approaches.

In summary, the proposed CNN–BiLSTM–Attention framework jointly learns spatial correlations, temporal dependencies, and contextual salience. By integrating these three aspects, the model provides a comprehensive solution to the challenges of predicting the remaining completion time of manufacturing orders. The integration of CNN for spatial feature extraction, BiLSTM for temporal sequence modeling, and Attention for dynamic feature selection results in a highly flexible and robust architecture. The proposed approach not only improves prediction accuracy and stability but also enhances the model ability to generalize across a wide variety of manufacturing scenarios, even when faced with diverse and noisy dataset.

## 5. Experimental Studies

### 5.1. Experimental Environment

**Figure 5:**
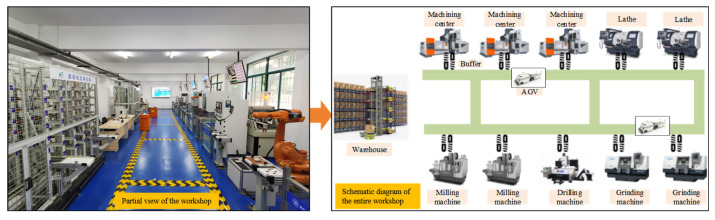
Manufacturing workshop scene.

The approach proposed in this paper was applied in an aerospace discrete manufacturing workshop to validate its feasibility and superiority. The workshop was already integrated with information systems such as MES, ERP, and WMS, and data collection devices like RFID were deployed on the equipment. The workshop consists of 10 machines, including 3 machining centers, 2 lathes, 2 milling machines, 2 grinding machines, and 1 drilling machine. Each machine was equipped with both in-buffer and out-buffer areas (as shown in Figure 5). Additionally, each part undergoes a quality inspection upon completion of machining. The workshop primarily processes five types of parts: pipe, shaft, end cover, plate, and bracket. The processing routes and times for each type of part were shown in Table 2. To improve the analysis of the experiments, this paper performed a predictive analysis on 27,832 data from 60 production orders in the workshop. Six network models, including BP, DBN, CNN, BiLSTM, CNN-LSTM and CNN-BiLSTM, were introduced for comparative analysis with the proposed model. Specific experimental details are provided in Section 5.2, Section 5.3 and Section 5.4. The algorithm was implemented in Python 3.9, using the TensorFlow 2.9.0 and Keras frameworks 2.9.0. The tests were run on a computer with 32 GB of RAM and an AMD Ryzen 7 4800H processor, with a clock speed of 2.90 GHz. Table 3 shows the parameters of the CNN–BiLSTM–Attention model.

### 5.2. Analysis of Experimental Results

In total, 27,832 pieces of workshop data were collected from the actual production process in this paper. The sample instances are shown in Table 4. These data include real-time operational records, such as machine states, process durations, and completion timestamps, which were aligned with the corresponding planned completion times extracted from the production schedule. First, the raw data were preprocessed to handle outliers and missing values, ensuring the consistency and reliability of the dataset. Then, the weight values of the features were calculated, and feature selection was performed based on their importance to the remaining completion time prediction. As a result, 558 features with the highest relevance were retained to construct the final feature dataset.

To ensure robust model generalization, the dataset was randomly shuffled and divided into training and testing sets at a 7:3 ratio. The model was trained on the training set and validated using the testing set by comparing the predicted completion times with the planned completion times from the production schedule, thereby quantifying the deviation between actual and baseline production progress. This comparison process verifies that the proposed CNN–BiLSTM–Attention-based prediction model can effectively capture the dynamic relationship between actual workshop performance and the planned production targets.

**Figure 6:**
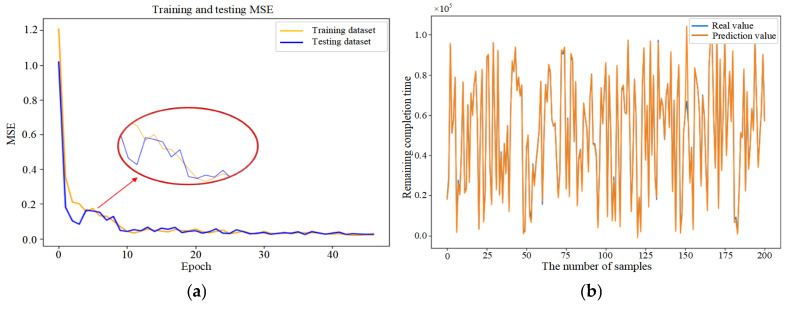

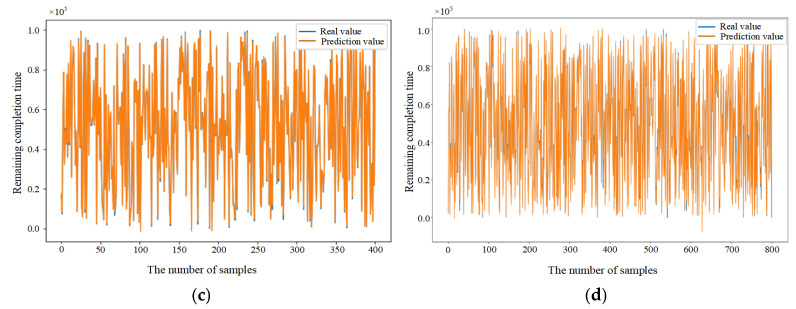
(**a**) Training process of the proposed model. (**b**) Prediction results based on 200 sample data. (**c**) Prediction results based on 400 sample data. (**d**) Prediction results based on 800 sample data.

Mean Squared Error (MSE) was introduced in this paper as the loss function (as shown in Equation (33)) to evaluate the discrepancy between actual and predicted values, thereby demonstrating the convergence of the CNN–BiLSTM–Attention model (as shown in Figure 6a). Initially, the prediction model failed to learn the relationships between features, resulting in substantial fluctuations despite a consistent decrease in the first 26 epochs. After 27 epochs, the model achieved similar error rates on both the training and testing datasets, with the MSE on the testing dataset converging to 0.056. The fluctuation in the convergence curve was significantly reduced, indicating that the model was successfully trained without overfitting.

In addition, in order to provide further validation of the robustness of the proposed model, experiments were conducted by randomly selecting 200, 400, and 800 data samples from the feature dataset. As demonstrated in Figure 6b–d, the proposed model consistently demonstrates excellent prediction performance when comparing the actual and predicted values for different sample sizes. This finding serves to confirm the generalization capability of the CNN–BiLSTM–Attention model.

(33) $$\mathrm{MSE}=\frac{1}{n}\sum_{i=1}^{n}{\left(y_{i}-y_{i}^{p}\right)}^{2}$$

where *n* denotes the number of samples, $y_{i}$ and $y_{i}^{p}$ denote the real value and predicted value of the *i*-th sample.

### 5.3. Comparative Analysis of Predictive Methods

**Figure 7:**
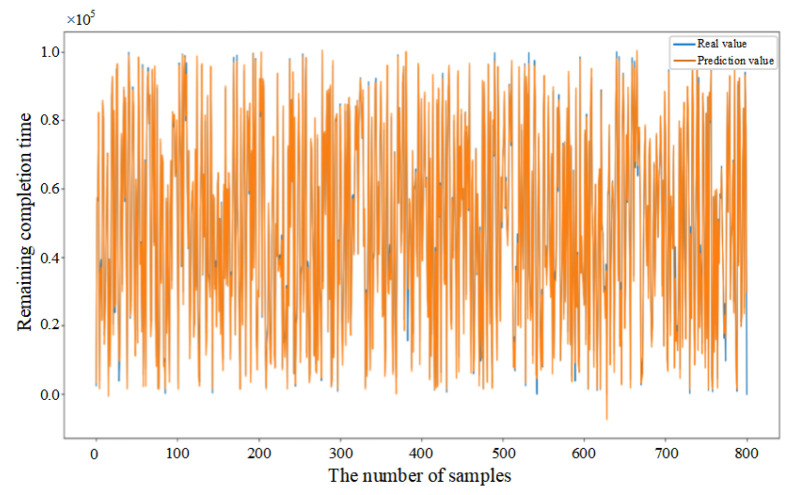
Prediction results of the CNN–BiLSTM–Attention model based on the sample data.

**Figure 8:**
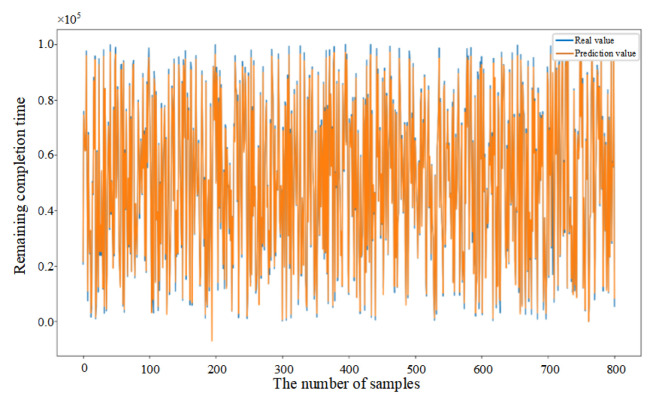
Prediction results of the CNN-BiLSTM model based on the sample data.

**Figure 9:**
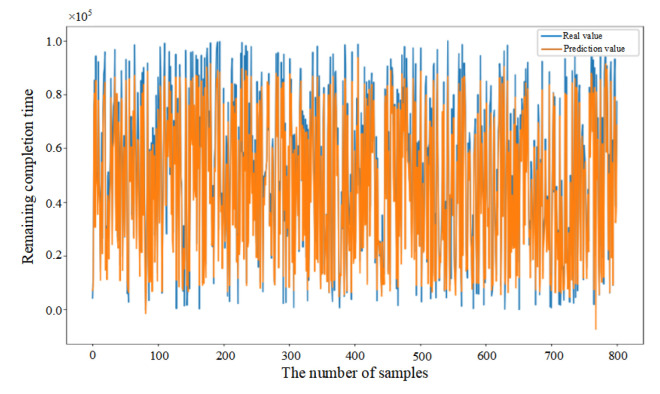
Prediction results of the CNN-LSTM model based on the sample data.

**Figure 10:**
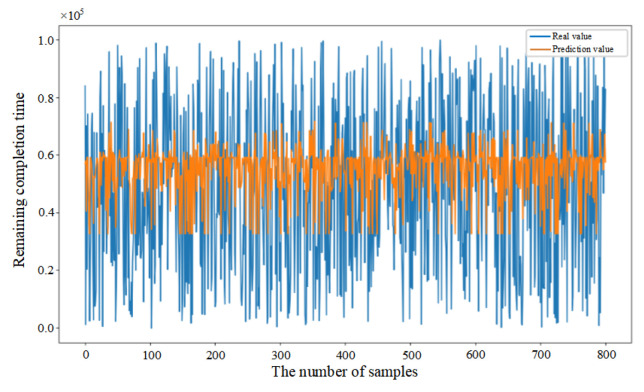
Prediction results of the CNN model based on the sample data.

**Figure 11:**
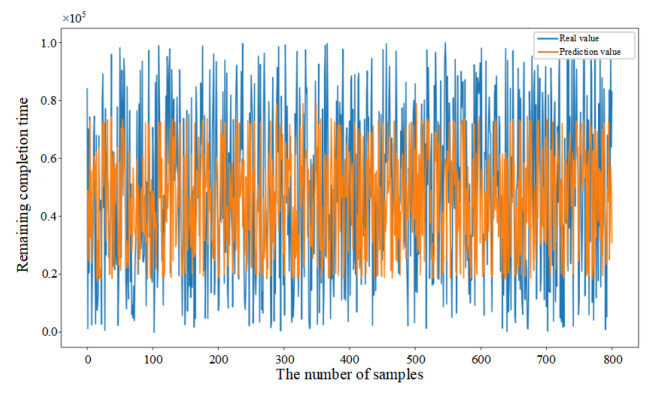
Prediction results of the BiLSTM model based on the sample data.

**Figure 12:**
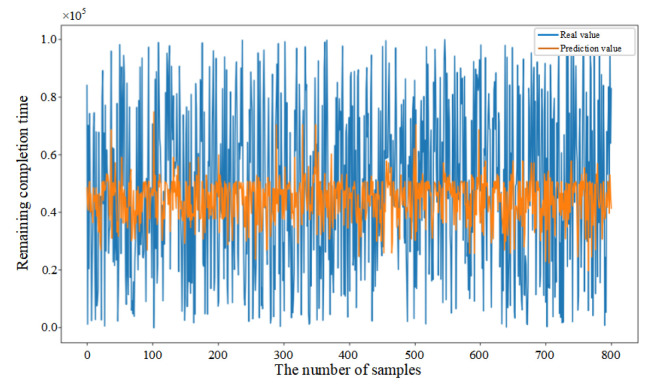
Prediction results of the BP model based on the sample data.

**Figure 13:**
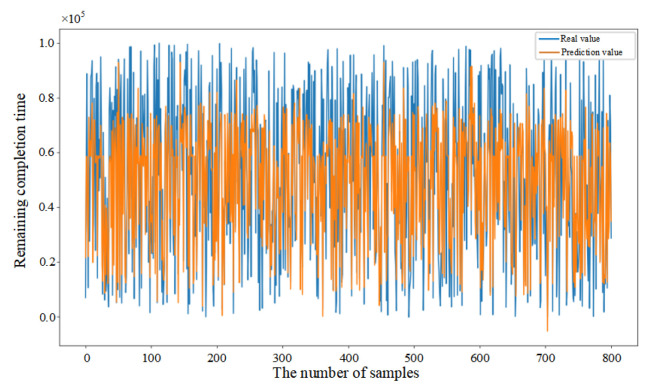
Prediction results of the DBN model based on the sample data.

To validate the superiority of the proposed method, comparative experiments were conducted using neural network models based on BP, DBN, CNN, BiLSTM, CNN-LSTM, and CNN-BiLSTM. In the experiments, 800 data samples were randomly selected. The prediction results for each model are shown in Figure 7, Figure 8, Figure 9, Figure 10, Figure 11, Figure 12 and Figure 13. The blue curve represents the actual order completion time, while the orange curve represents the predicted order completion time. When the predicted completion time aligns with the actual completion time, the orange curve overlaps the blue curve. When the completion times diverge, the orange curve exhibits errors relative to the blue curve. Larger errors indicate poorer prediction performance. The results in Figure 7, Figure 8, Figure 9, Figure 10, Figure 11, Figure 12 and Figure 13 reveal that Figure 7 barely shows the blue curve, indicating the CNN–BiLSTM–Attention model achieves remarkable prediction accuracy, nearly matching the predicted completion time with the actual completion time at 100%. Figure 8 shows a slightly more visible blue curve, representing the CNN-BiLSTM model with marginally lower prediction performance. Figure 9, Figure 10, Figure 11, Figure 12 and Figure 13 display a more prominent blue curve, signifying poorer prediction accuracy for the other models. Comparative analysis of different algorithms demonstrates that the proposed model exhibits the smallest prediction error between predicted and actual completion times, showcasing exceptional predictive accuracy. Additionally, the proposed method achieves outstanding computational efficiency, resulting in significantly reduced running time, further confirming the practical superiority for predicting order remaining completion time.

The comparative results presented in Figure 7, Figure 8, Figure 9, Figure 10, Figure 11, Figure 12 and Figure 13 clearly demonstrate that the proposed CNN–BiLSTM–Attention model achieves the best prediction performance across all evaluated methods. Unlike traditional models such as BP and DBN, and even deep learning variants like CNN, BiLSTM, and CNN-LSTM, the proposed model exhibits significantly lower error margins and stronger consistency with the actual completion times. The superior performance of the model is not only due to the combination of advanced modules, but more importantly, to the task-specific architecture design: the multi-kernel CNN effectively captures heterogeneous spatial patterns from manufacturing data, the BiLSTM module models complex bidirectional dependencies in temporal production sequences, and the attention mechanism adaptively highlights critical time steps that have a disproportionate impact on order completion. These components are cohesively integrated to form a robust and interpretable framework, well aligned with the dynamic, noisy, and multidimensional nature of real-world manufacturing environments.

**Figure 14:**
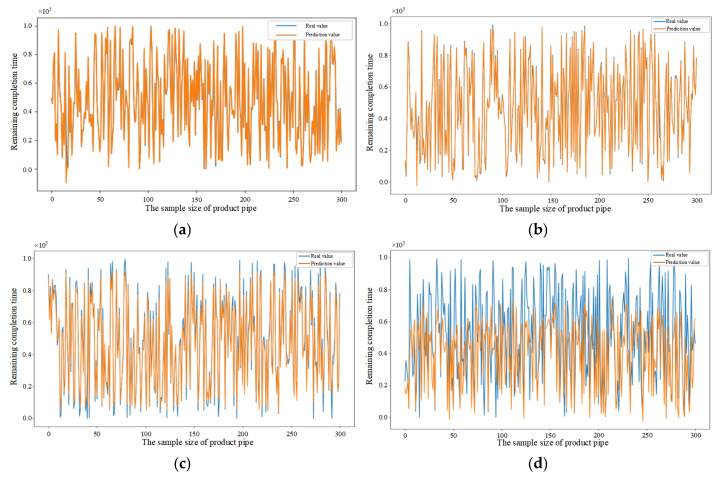

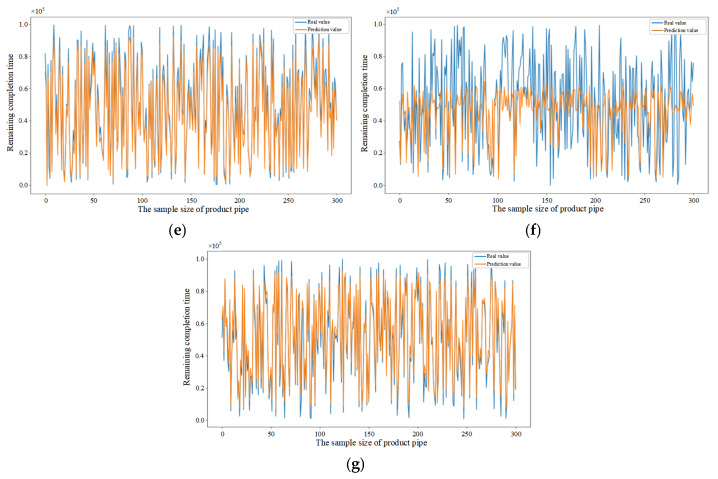
(**a**) Prediction results of CNN–BiLSTM–Attention model based on pipe sample data. (**b**) Prediction results of CNN-BiLSTM model based on pipe sample data. (**c**) Prediction results of CNN-LSTM model based on pipe sample data. (**d**) Prediction results of CNN model based on pipe sample data. (**e**) Prediction results of BiLSTM model based on pipe sample data. (**f**) Prediction results of BP model based on pipe sample data. (**g**) Prediction results of DBN model based on pipe sample data.

**Figure 15:**
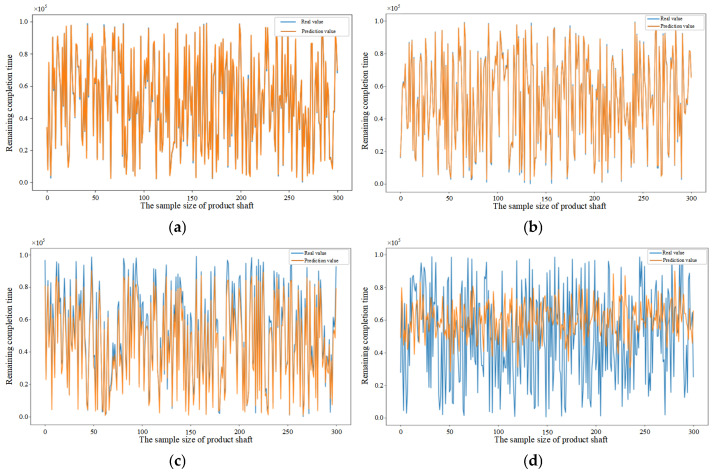

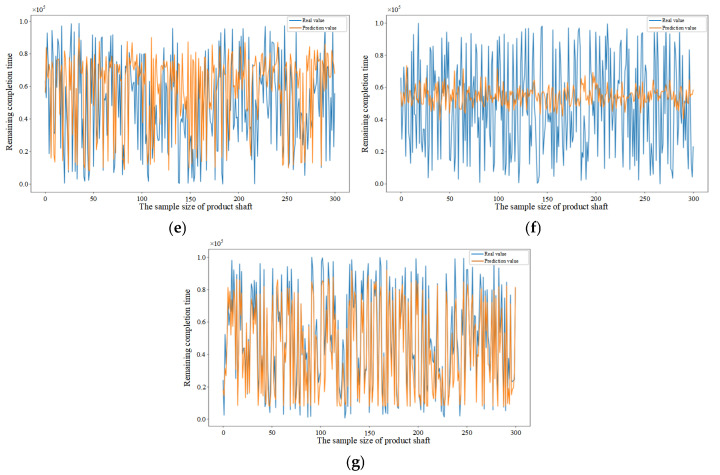
(**a**) Prediction results of CNN–BiLSTM–Attention model based on shaft sample data. (**b**) Prediction results of CNN-BiLSTM model based on shaft sample data. (**c**) Prediction results of CNN-LSTM model based on shaft sample data. (**d**) Prediction results of CNN model based on shaft sample data. (**e**) Prediction results of BiLSTM model based on shaft sample data. (**f**) Prediction results of BP model based on shaft sample data. (**g**) Prediction results of DBN model based on shaft sample data.

**Figure 16:**
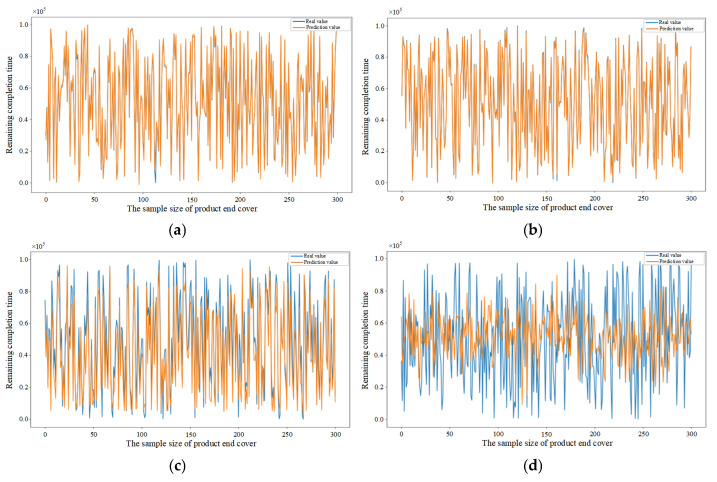

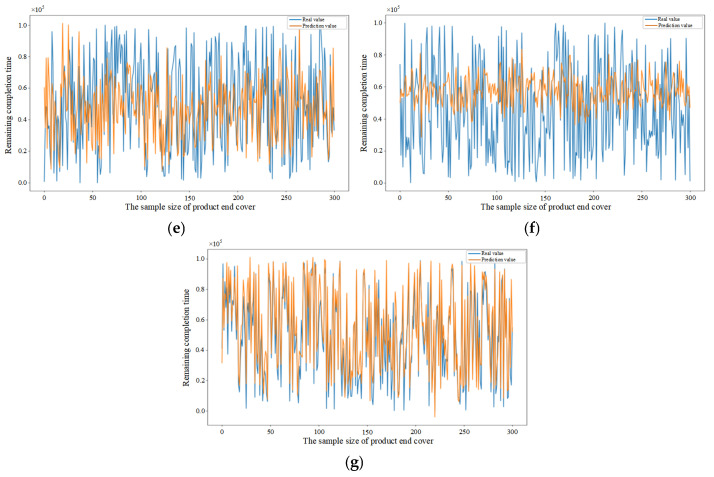
(**a**) Prediction results of CNN–BiLSTM–Attention model based on end cover sample data. (**b**) Prediction results of CNN-BiLSTM model based on end cover sample data. (**c**) Prediction results of CNN-LSTM model based on end cover sample data. (**d**) Prediction results of CNN model based on end cover sample data. (**e**) Prediction results of BiLSTM model based on end cover sample data. (**f**) Prediction results of BP model based on end cover sample data. (**g**) Prediction results of DBN model based on end cover sample data.

To further demonstrate the generalization capability of the proposed model across diverse manufacturing settings, experimental data were collected from three distinct types of parts (pipe, shaft, and end cover) within different manufacturing orders, as detailed in Table 5. The selection of these parts is based on their distinct processing routes, which necessitates the utilization of different machines for processing and thereby represents varying manufacturing environments. The feature datasets collected during the processing of the pipe, shaft, and end cover were filtered and analyzed. The randomly selected test samples were 300 in each dataset. Subsequently, the order remaining completion time prediction was performed using the CNN–BiLSTM–Attention, CNN-BiLSTM, CNN-LSTM, CNN, BiLSTM, BP, and DBN, respectively, and the results are shown in Figure 14, Figure 15 and Figure 16. Figure 14a–g illustrate the results of the pipe part predicted by different models, Figure 15a–g illustrate the results of the shaft part predicted by different models and Figure 16a–g illustrate the results of the end cover part predicted by different models. The results demonstrate that the model proposed in this paper exhibits superior prediction accuracy and enhanced generalization capability, as evidenced by the validation of the model with randomly selected test samples in diverse manufacturing settings.

Figure 14a–g compare the performance of different prediction models in the order remaining completion time prediction for pipe part.

Figure 15a–g compare the performance of different prediction models in the order remaining completion time prediction for shaft part.

Figure 16a–g compare the performance of different prediction models in the order remaining completion time prediction for end cover part.

### 5.4. Performance Evaluation of Predictive Methods

Additionally, in order to further validate the superiority of the CNN–BiLSTM–Attention model in predicting the order remaining completion time, the prediction results of different models were evaluated using four metrics: Mean Absolute Error (MAE), Root Mean Squared Error (RMSE), Mean Absolute Percentage Error (MAPE), and the coefficient of determination (R^2^).

The RMSE emphasizes larger errors, and the value will be higher if the model has a large deviation in order remaining completion time prediction. In manufacturing workshop, accurate time prediction is essential to optimize resource scheduling and reduce production bottlenecks, as large errors can mean serious problems with production planning and lead to wasted resources. The MAE directly calculates the average error size, with equal penalties for small and large errors. In the production process, the MAE reflects the overall accuracy of the forecast results. A smaller MAE means that the forecast is closer to the actual situation, thus contributing to more accurate production planning. MAPE is a measure of the relative error of a forecast and is particularly suitable for forecasts where the target is positive. It expresses the magnitude of error as a percentage, which is easy to understand and compare. In production prediction on the manufacturing workshop, MAPE can inform manager the model predictions accuracy. R^2^ measures how well a prediction model captures the variability in the data. The closer the R^2^ value is to 1, the better the model can explain the data variations. In workshop production, R^2^ serves as an indicator of the model overall predictive power. A higher R^2^ value indicates that the model is more effective, thereby improving the efficiency of production planning. The Equations are shown as follows:

(34) $$\mathrm{MAE}=\frac{1}{n}\sum_{i=1}^{n}\left|y_{i}-y_{i}^{p}\right|$$

(35) $$\mathrm{RMSE}=\sqrt{\frac{1}{n}\sum_{i=1}^{n}{\left(y_{i}-y_{i}^{p}\right)}^{2}}$$

(36) $$\mathrm{MAPE}=\frac{100}{n}\sum_{i=1}^{n}\frac{\left|y_{i}-y_{i}^{p}\right|}{y_{i}}\%$$

(37) $$R^{2}=1-\frac{\sum_{i=1}^{n}{\left(y_{i}-y_{i}^{p}\right)}^{2}}{\sum_{i=1}^{n}{\left(y_{i}-\bar{y}\right)}^{2}}$$

where *n* denotes the number of test data samples, $y_{i}$ and $y_{i}^{p}$ denote the actual and predicted values of the *i*-th sample, and $\bar{y}$ denotes the processed average of the *i*-th sample.

All models were trained and tested on the same dataset under identical experimental settings, the evaluation metrics for different predictive models are shown in Table 6. R^2^ and MAE are the primary metrics for predicting the remaining order completion time. R^2^ reflects the overall goodness-of-fit between the predicted and actual values, while MAE indicates the average deviation between the predicted and actual values, providing an accurate measure of the prediction error. Compared with CNN-BiLSTM, CNN-LSTM, CNN, BiLSTM, BP, and DBN models, the CNN–BiLSTM–Attention model achieves an R^2^ of 0.983 and an MAE of 0.011, both significantly outperforming other models. Additionally, the RMSE and MAPE metrics further confirm its superior performance, with values of 0.057 and 17.352%, respectively.

The experimental results show that the structural design of the CNN–BiLSTM–Attention model is a carefully crafted integration tailored to the nature of complex, multi-source manufacturing data. The empirical results strongly support the technical value and structural contribution of the proposed model in industrial order completion time prediction tasks. Overall, the model presented in this paper meets the requirements of most scenarios in a manufacturing workshop and accurately predicts the remaining completion time of orders.

## 6. Conclusions

This paper proposes a CNN–BiLSTM–Attention-based hybrid deep learning optimization model for predicting the remaining completion time of manufacturing orders. First, the anomalous and missing data in the original dataset are processed through data preprocessing (e.g., capping method, median imputation), so as to construct an effective feature dataset for predicting the remaining completion time. In addition, by combining CNN for spatial feature extraction, BiLSTM for bidirectional temporal modeling, and an Attention mechanism for dynamic feature weighting, the model effectively captures complex spatiotemporal dependencies in production data. Finally, experimental results based on real workshop data demonstrate that the proposed model achieves a prediction accuracy of 0.983, outperforming traditional approaches. The proposed model is suitable for real-time deployment via edge devices and can be integrated into smart production systems for dynamic monitoring and scheduling. With continuous data updates, the framework supports incremental training and adaptive optimization, providing a robust solution for intelligent manufacturing environments.

The proposed CNN–BiLSTM–Attention model was developed and validated for discrete manufacturing workshops, specifically in the context of aerospace part production. This use case is representative of high-mix, low-volume discrete manufacturing systems characterized by complex routing, order-driven workflows, and dynamic process changes. Therefore, the proposed approach is well suited for discrete industries, where production environments share similar scheduling characteristics. Nevertheless, different industries exhibit distinct scheduling mechanisms, process dependencies, and value-chain interactions. Although the underlying modeling framework is transferable, applying it to continuous or process-oriented industries would require additional adaptation of data features and model structures to reflect distinct scheduling mechanisms and process dependencies.

In future research, consider that as the data volume increases, especially for real-time predictions in large workshop environments with multiple orders, the CNN–BiLSTM–Attention model may encounter memory and computational bottlenecks. Therefore, in order to adapt to large-scale datasets, on the one hand, distributed computing platforms (e.g., GPU clusters, TPUs) can be considered to accelerate the model training process. On the other hand, model compression techniques (e.g., pruning, quantization, and knowledge distillation) can be investigated to improve the inference speed and computational efficiency of the model by reducing its size and complexity, thus better adapting to the real-time prediction needs of large-scale workshop production.

## Figures and Tables

**Table 1:** Workshop characteristic dataset.

Feature Type	Feature Description
Features of WIP	The number of operations *N_i_* required for WIP *i*
	The total processing time *T_i_* of WIP *i*
	The longest processing time *T_di_* required for work in progress *i* on machine *d*
	Quality status *Q_i_* of WIP *i*
	The sum of the waiting times *W_ti_* of machine m on the processing path of WIP *i*
	The total quantity of WIP *S_i_*
	Work in process *i* completes processing process route *R_i_*
Features of machines	Processed part *P_i_* on machine *d*
	The number of parts to be processed *C_di_* queued up on machine *d*
	The total number of jobs *T_d_* currently being processed on machines 1, …, *d*
	The total remaining workload *R_d_* of the machine 1, …, *d* processing operation
	The total processing time *T_d_* of machine 1, …, *d*
Features of orders	Order *O* status *S_o_* before arriving at the workshop
	Priority *P_o_* of order *O*
	Quantity per order *O_s_*
	Order 1, …, *n* total workload *O_n_*
	Average circulation time *F_t_* of orders 1, …, *n*
	The sum of remaining processing times *O_t_* for all orders in the workshop

**Table 2:** Part process route information.

Part Number	Part Type	Process Route	Processing Time (s)
1	Pipe	Lathing-Drilling-Grinding	26-15-12
2	Shaft	Lathing-Milling-Grinding	18-16-12
3	End cover	Machining center-Grinding	26-28
4	Plate	Machining center-Drilling-Grinding	30-26-29
5	Bracket	Machining center-Milling-Grinding	24-19-27

**Table 3:** CNN–BiLSTM–Attention model parameters.

Model	Parameters	Value
CNN–BiLSTM–Attention	Number of convolution kernels in the first convolutional layer	256
	Number of convolution kernels in the second convolutional layer	256
	Number of neurons in the BiLSTM layer	128
	Number of neurons in the first fully connected layer	128
	Number of neurons in the second fully connected layer	1
	Number of iterations	1000
	Learning rate	0.001
	Dropout rate	0.2
	Activation_CNN	ReLU
	Activation_BiLSTM	tanh
	Activation_Attention	tanh

**Table 4:** Sample instances of order remaining completion time.

Category	Data Characterization	Data Examples
WIP status	Number of processes	2, 3, 2, 2, 3, …
	Total processing time	53, 46, 70, 54, 46, 85, …
	Maximum processing time	26, 28, 30, 36, 41, …
	Product quality status	0, 0, 0, 3, 0, 1, …
	Process route	2, 3, 2, 3, 3, 2, …
	Waiting time	12, 20, 14, 16, 36, …
	Total WIP	252, 160, 350, 340, 150, …
Machine status	Waiting queue length (per machine)	3, 2, 2, 1, 0, 5, …
	Remaining processing tasks	10, 12, 8, 6, 13, …
	Total processing time	230, 195, 250, 180, …
	Current state	0, 0, 3, 1, 1, 0, 2, …
Order status	Number of orders	20, 15, 30, 20, …
	Number of parts per order	8, 10, 12, 15, 30, …
	Order priority	1, 2, 3, 1, 1, …

**Table 5:** Order data under different manufacturing settings.

Manufacturing Setting	Order	Part	Number
Manufacturing Setting 1	Order 1	Pipe	50
	Order 2	Pipe	30
	Order 3	Pipe	40
Manufacturing Setting 2	Order 4	Shaft	60
	Order 5	Shaft	20
	Order 6	Shaft	30
Manufacturing Setting 3	Order 7	End cover	50
	Order 8	End cover	40
	Order 9	End cover	40

**Table 6:** Testing results for different models.

Model	R^2^	MAE	RMSE	MAPE
DBN	0.635	0.022	0.215	53.274%
BP	0.386	0.351	0.362	133.413%
BiLSTM	0.612	0.024	0.183	44.116%
CNN	0.469	0.229	0.268	114.086%
CNN-LSTM	0.728	0.0216	0.135	52.426%
CNN-BiLSTM	0.967	0.015	0.061	21.898%
CNN–BiLSTM–Attention	0.983	0.011	0.057	17.352%

## Data Availability

The data used during this paper are available from the corresponding author upon reasonable request.
